# Optimization of Printing Parameters for Digital Light Processing 3D Printing of Hollow Microneedle Arrays

**DOI:** 10.3390/pharmaceutics13111837

**Published:** 2021-11-02

**Authors:** Essyrose Mathew, Giulia Pitzanti, Ana L. Gomes dos Santos, Dimitrios A. Lamprou

**Affiliations:** 1 School of Pharmacy, Queen’s University Belfast, Belfast BT9 7BL, UK; emathew01@qub.ac.uk (E.M.); giulia.pitzanti@qub.ac.uk (G.P.); 2 Pharmaceutical Sciences, R&D BioPharmaceuticals, AstraZeneca, Granta Park, Cambridge CB21 6GH, UK; ana.gomesdossantos@astrazeneca.com

**Keywords:** hollow microneedles, transdermal drug delivery, 3D printing, additive manufacturing, digital light processing, emerging technologies

## Abstract

3D printing is an emerging technology aiming towards personalized drug delivery, among many other applications. Microneedles (MN) are a viable method for transdermal drug delivery that is becoming more popular for delivery through the skin. However, there is a need for a faster fabrication process with potential for easily exploring different geometries of MNs. In the current study, a digital light processing (DLP) method of 3D printing for fabrication of hollow MN arrays using commercial UV curable resin was proposed. Print quality was optimised by assessing the effect of print angle on needle geometries. Mechanical testing of MN arrays was conducted using a texture analyser. Angled prints were found to produce prints with geometries closer to the CAD designs. Curing times were found to affect the mechanical strength of MNs, with arrays not breaking when subjected to 300 N of force but were bent. Overall, DLP process produced hollow MNs with good mechanical strength and depicts a viable, quick, and efficient method for the fabrication of hollow MN arrays.

## 1. Introduction

The skin is the largest organ in the human body and covers an area of ~1.8 m^2^ in the average person [1]. The primary function of the skin is to act as a barrier to the entry of harmful substances such as pathogens from the environment into the body. The outermost layer of the skin, known as the stratum corneum (SC), is around 10–20 µm-thick. The SC serves as the primary barrier to permeation through the skin. The skin can protect against the permeation of ultraviolet (UV) radiation, pathogens, allergens, and prevents the loss of moisture and nutrients from the body [2]. The skin provides an ideal site for delivery of topical therapeutic agents, mainly for the treatment of dermatological conditions including microbial infections, psoriasis, and eczema [3]. However, in reality, the skin is impermeable to a vast array of drug compounds due to its high barrier properties. When treatment is applied topically to the skin, the drugs can have a local effect on the skin or be absorbed through the skin where they can exert a systemic effect. Drugs aimed to be employed in skin drug delivery must contain specific physiochemical properties including low molecular weight below 500 Dalton, low melting point (<250 °C), high lipophilicity, and a log *p* value between 1 and 5 [2]. Transdermal Drug delivery (TDD) describes the transfer of an active pharmaceutical ingredient (API) through the skin into the dermal microcirculation for their absorption where they can have a systemic effect [3].

Microneedle (MN) arrays have been developed to effectively overcome the SC barrier. MNs are small needles, ranging from a few microns to up to 2 mm in height, which are able to breach the SC without reaching the nerve endings in the dermal tissues, allowing for pain-free drug delivery [4,5]. MNs provide the convenience and safe pain-free application provided by a transdermal patch whilst maintaining the efficiency and delivery into systemic circulation of hypodermic needles [6]. Polymeric MNs are often fabricated using the micromoulding technique, which involves the pouring of liquid polymeric material into a laser-engineered silicon MN mould and removal of air by use of vacuum or centrifugation, followed by drying and removal from the mould, which can take over 24 h for the full process [7]. Hollow MNs in particular are a viable method for the delivery of drugs through a transdermal route. Hollow MNs work by creating microchannels in the skin when inserted, allowing continuous delivery of liquid drug formulations through these channels. The driving force of the drug from the MN patch into the skin can vary, obtaining pressure through a syringe system, pump, or microfluidic chip. One advantage of hollow MNs is the ability to deliver larger capacities of drugs through the skin in comparison with their counterparts of solid, dissolving, and coated MNs [3]. Hollow MNs are typically limited by their mechanical strength due to the presence of a bore through the centre of the MN. Hollow MNs have been fabricated using ceramics, metal, silicon, and glass [8,9,10]. Recently, biocompatible polymers have more commonly been used for fabrication of MNs as they are more cost efficient, can be disposed of safely, and can be tailored for controlled-release profiles. Hollow MNs can be fabricated through a range of techniques including micromoulding and micromachining [11]. These processes can often be time consuming and require multiple fabrication steps.

3D printing (3DP) allows for a customisable design of MN arrays, making it a convenient and flexible approach for the fabrication of MN arrays [12]. 3DP can cater for differences in skin thickness and hydration, which are factors affecting the drug delivery capabilities of transdermal systems [13]. 3DP MNs will aid the movement towards personalised medicine as designs and drug loading can be modified based on the individual [14]. 3DP has been used for the creation of female moulds for the production of MNs; however, there are limitations in that for any new changes to needle geometries, new moulds would need to be created [15]. 3DP of hollow MNs has not been widely explored due to the limited resolution capabilities of printers. 2-photon-polymerisation (2PP) is a high-resolution 3DP technique; however, it can be very expensive and take longer to print models than other types of printers such as Stereolithography (SLA) or Fused Deposition Modelling (FDM) [16,17]. 2PP methods outlined in research often involve multiple fabrication steps, which can be time consuming [18]. Other resin-based printing techniques that have been shown to form hollow MN arrays include using SLA, which has shown to be a feasible method for additive manufacture (AM) [19,20].

In this article, we propose a 3DP fabrication method of hollow MNs using the Digital Light Processing (DLP) 3DP technique. DLP differs from other resin-based printing as it uses UV light through a projector to cure resin layer-by-layer according to the computer aided design (CAD). The use of a projector means that each full layer is cured in one go allowing for faster print times in comparison with SLA, for which speed is dependent on laser point size [21]. DLP printers can also print to the micron scale, allowing it to be a suitable method for production of MNs. Although hollow MNs have been printed successfully in previous studies using SLA, we hope to explore the DLP technique in more detail due to its ability to rapidly manufacture high-resolution prints at faster times than SLA. This manuscript explores the optimisation of design, printing parameters, and postprinting steps to maximise the printing quality and sharpness of MN arrays. We will explore the printability of three resin-based 3DP techniques (DLP, SLA, UV LCD), which to the best of authors knowledge, is the first time this comparison has been reported in literature.

## 2. Materials and Methods

### 2.1. Materials

Form 2 MN arrays were fabricated using Class I biocompatible Dental SG resin (Formlabs, Ripon, UK). Inkspire MN arrays were fabricated using white resin (Zotrax, Olsztyn, Poland). Asiga Max UV MN arrays were fabricated using PLasGRAY resin (Asiga, Alexandria, Australia). Isopropyl Alcohol was purchased from Sigma-Aldrich (St. Louis, MO, USA). Parafilm^®^ M was purchased from Bemis Company (Neenah, WI, USA).

### 2.2. Design and Manufacture of MNs

#### 2.2.1. DLP Printing

**Figure 1:**
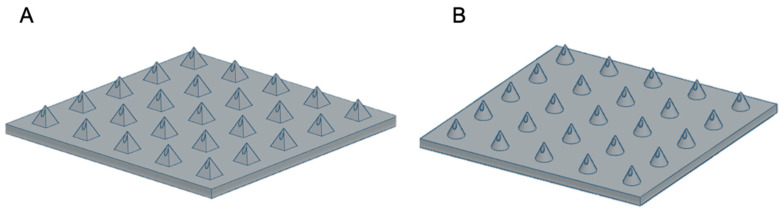
Computer-Aided Design of PyMN (**A**) and CoMN (**B**).

MN arrays were created online using free-access CAD software (TinkerCAD, USA). MN arrays were designed with a 15 × 15 × 0.5 mm base plate; needles were 1 mm in height for both Conical microneedles (CoMN) and Pyramidal microneedles (PyMN). CoMN had 1 mm base diameter and PyMN had 1 × 1 mm base and 0.25 × 0.25 mm bore diameters for both shapes, as shown in Figure 1. Designs were converted to .stl files and uploaded to the slicing software Asiga composer, imported MN designs were positioned at an angle of 0° and supports were added. Designs were printed at a resolution of 0.025 mm using PlasGRAY resin (Asiga, Alexandria, Australia). MNs were printed using the Asiga Max UV printer (Asiga, Alexandria, Australia). After printing was complete, the MN array was removed from the build plate and supporting structures were removed. The MN arrays were then immersed in Isopropyl Alcohol (IPA) and sonicated using the Ultrawave QS12 Ultrasonic Bath (Dorset, UK) for 3 min in order to remove any excess Plasgray from the model. After sonication, the arrays were removed from the IPA and left to air dry. After prints were dried, the prints were placed into a 385-nm UV chamber (Asiga Flash, Melbourne, Australia), where they cured for 20 min as recommended by the printer manufacturer.

#### 2.2.2. SLA 3D Printing

A Form 2 SLA-based 3D printer was used for the printing of MNs for comparison purposes. The same MN designs shown in Figure 1 were imported onto the slicing software associated with the Form 2 printer, Preform (Formlabs, Somerville, MA, USA). MN designs were oriented at a 45° angle and supports added with a touchpoint size of 0.4 mm and density of 1. The processed designs were then exported as .stl files to the printer and printed using Dental SG resin at the resolution of 0.050 mm. Post printing, the MN arrays were removed from the printing platform and detached from the supporting structures. The arrays were then washed in IPA to remove any excess unpolymerized resin and cured under UV light at 40 °C for 60 min using an UV-A chamber (MeccatroniCore BB Cure Dental station, North Yorkshire, UK) at a wavelength of 405 nm.

#### 2.2.3. UV LCD Printing

A Zortrax Inkspire UV LCD printer (Zotrax, Olsztyn, Poland) was used to print MN arrays for comparison purposes with the Asiga MAX 3D prints. The .stl files were uploaded on the Z-suite slicing software (Zotrax, Olsztyn, Poland) of the Inkspire printer, supporting structures were added, and the model was sent to the Inkspire 3D printer and printed at a resolution of 0.025 mm. Models were printed using UV White/Ivory Resin (Zortrax, Olsztyn, Poland). After printing was completed, the print was removed from the build plate, placed in an IPA bath, and sonicated for 2 min using Ultrawave QS12 Ultrasonic Bath to remove any excess resin remaining on the surface of the prints. After prints were dry, the prints were cured for 30 min under UV light.

### 2.3. Imaging of 3D Printed MNs

Printed MN arrays were analysed using Scanning Electron Microscopy (SEM), using Hitachi TM3030 equipment (Tokyo, Japan). The arrays were viewed in the Energy Dispersive X-Ray (EDX) condition. The MN arrays were mounted onto the sample holder with double-sided carbon tape, placed into the SEM chamber, and analysed under vacuum. Measurements of base diameter and tip size were recorded. MN heights were recorded using optical light microscopy Leica EZ4D (Leica Microsystems, Milton Keynes, UK). A Keyence VHX-700F Digital Microscope (Keyence, Osaka, Japan) was also used to visualise the MNs, allowing for 3D reconstruction of the MN array structures.

### 2.4. Angled Prints for Print Optimisation

15 × 15 × 1 mm base with 1 × 1 mm solid needles as well as 1 × 1 mm needles with 0.25 × 0.25 mm bore were printed in both CoMN and PyMN shapes. To analyse the effect of print angle on the needle geometry, in the preprocessing Composer software of the Asiga Max, the MN arrays were angled at 0°, 15°, 30°, 45°, 60°, 75°, and 90° from the base plate. The arrays were printed in triplicate for each angle using the Asiga Max UV 3D printer. After printing, each MN array was analysed using SEM and Light Microscopy and measurements of base width of needles, tip size, and needle heights were recorded.

### 2.5. Parafilm Insertion Tests

Depth of insertion of MN arrays were analysed using parafilm insertion tests as developed by Larreneta et al. [22]. Parafilm was cut into 10 squares, approx. 2 × 2 cm each, and laid on top of each other to create model skin. Each layer of parafilm was approx. 127 µm in height. Therefore, the 10 layers created a 1.27-mm skin model. A TA.XTPlus Texture Analyser (Stable Micro Systems, Surrey, UK) was used to exert chosen forces on the MNs. A cylindrical probe was used to exert force on the MN array. The probe moved down at a speed of 1.19 mm/s until a pre-set force was reached. The force was exerted for 30 s and then the MN array was removed from the Parafilm layers. Layers were separated and the number of holes produced in each layer was analysed using light microscopy.

### 2.6. Mechanical Testing of MN Arrays

To assess the mechanical strength of the MN arrays at various curing times—0, 10, 20, and 30 min—fracture testing using the Texture analyser was performed as outlined by Donnelly et al. [7]. Briefly, MN arrays were attached to metal probe using adhesive tape. The texture analyser was set to compression mode and the metal probe with MN array attached was lowered towards an aluminium block at a speed of 0.5 mm/s until a force of 300 N was exerted. Images of MNs and needle heights were measured before and after mechanical fracture testing using light microscope. A force displacement graph was produced to quantify the fracture force of the needles. Percentage in height reduction was calculated using the following Equation (1):

(1) $$\%\;Height\;Reduction=\frac{H_{a}-H_{b}}{H_{a}}$$

where *H_a_* = Height before mechanical testing, *H_b_* = Height after mechanical testing.

### 2.7. Statistical Analysis

Quantitative data was expressed a mean ± standard deviation, *n* = 3. One-Way Analysis of Variance was used for statistical testing, with *p* < 0.05 considered to be statistically significant.

## 3. Results and Discussion

### 3.1. Comparison of Resin-Based Printers

**Figure 2:**
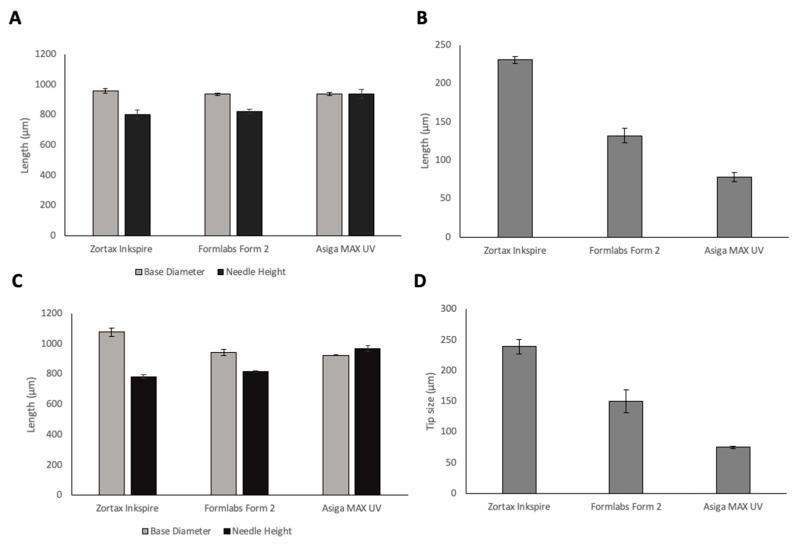
Base diameters and needle heights (**A**) and tip size (**B**) of 15 × 15 × 1 mm base 1 mm PyMN1 mm pyramidal (Py) needles; base diameters and needle heights (**C**) and tip size (**D**) of 15 × 15 × 1 mm base 1 mm conical (CoMN) needles printed using 3 different resin-based 3D printers.

To investigate the resolution capabilities of the printers, MN arrays were printed using three different resin-based 3D printers, a summary of the printers and their advantages and disadvantages are shown in Table 1. The needle geometries of printed MN arrays using the three different printers are shown in Figure 2. All printers were able to produce protruding needles. When looking at base diameter, LCD print has the closest value to the design geometry of 1000 µm. However, DLP print had the optimal needle height of 935.8 µm in comparison with 819.3 µm for Form 2 and 802 µm for LCD prints. Needle height is a critical parameter that determines insertion depth of MNs into the skin; therefore, it is essential to choose the printer that provides prints closest to the design geometry of the needle heights. DLP printer had a significantly higher needle height (*p* < 0.05) in comparison with the LCD and SLA printers, and therefore, presented optimal needle height. DLP prints had a significantly smaller tip size than SLA and LCD prints (*p* < 0.05) for both CoMNConical (Co) and Pyramidal (PyMN). Tip size is critical for the insertion capability of the MNs. Indeed, increased tip sharpness results in a lower insertion force required [23]. Therefore, when looking at the critical parameters of needle height and tip size, DLP produced prints that had the optimal geometry.

**Figure 3:**
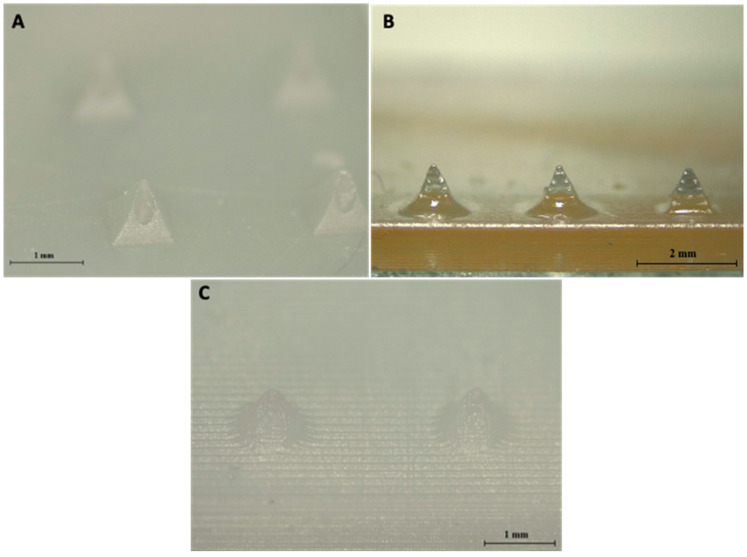
Hollow MN arrays printed using DLP (**A**), SLA (**B**), and LCD (**C**).

**Figure 4:**
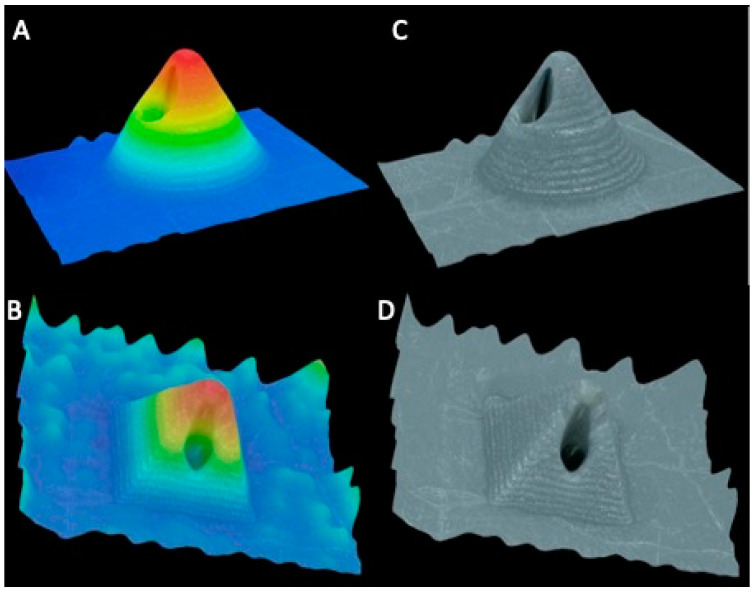
VH-700F digital microscope images of 3D reconstruction of MNs with colour scale, CoMN (**A**) and PyMN (**B**), and without colour scale, CoMN (**C**) and PyMN (**D**).

After printing hollow designs using the three different resin-based printers, only the DLP 3D printer produced needles with a visible bore running through each needle, as seen in Figure 3. Hollow MN arrays printed using DLP can also be seen clearly in the 3D reconstruction imaging, shown in Figure 4. LCD print was printed with a resolution of 25 µm, same as DLP; however, it is evident that there is a clear difference in the resolution capabilities of the printers between the DLP method and LCD method of printing, which can also be due to the material used. The material used in each type of printing will also have an influence on the print quality of the final print. As different materials were used in each printer, a direct comparison cannot be made between printer type and print quality. Dental SG resin used in SLA printer had an optimal resolution of 50 µm, which was not able to create a MN array with visible holes present. However, the minimum layer height of the printer is 25 µm; so, the material used may have resulted in a lower resolution print, resulting in no hole being present. One previously published paper outlined the creation of hollow MNs using the same printer and print material, with successful prints having visible bores present [19]. Therefore, there could be a difference in printing parameters input that resulted in no bore being present in the printed MN in our studies; factors such as build tray quality, print speed, and design software used could have influenced the quality of the prints. For comparison of the printers, prints were not oriented at an angle during printing; however, Economidou et al. printed at an angle of 45°. Further testing of angled prints using the SLA printer were not tested as DLP produced needles with bores without orienting the print at an angle and also printed the same design in a shorter period of time [19]. It can be concluded that the used DLP printer had the better resolution capabilities tested. Although a direct comparison cannot be made between the printers due to different materials, this method helps to identify the printer with the greatest potential for creating hollow 3DP microneedles.

### 3.2. Printing Capabilities of DLP Printer

In order to evaluate the printing capabilities of the printer, solid MNs with heights and diameters of 800 µm, 600 µm, 400 µm, and 200 µm were printed at a resolution of 0.025 mm and analysed using SEM. The 800-μm PyMN were printed successfully and the needles were well-defined, as shown in Figure S1. With the 800-μm CoMN, there were no definitive needles present, but this may be due to an error during the printing process as the lower 600 μm-height CoMNs were printed successfully. The 600-μm CoMNs were well-defined and printed well. In both the 400 μm CoMN and PyMN, it is visible that the needles are beginning to lose sharpness, as the top layer appears wider and flatter in comparison with the 800- and 600-μm needles due to fewer layers creating the required height. The 200-μm needles were not printed well in either of the shapes; only 2 layers appear on the needles so there is no sharpness or definitive shape to the needles. It can be noted with the 200-μm PyMN that the top layer on one of the needles has been printed beside the base, this shows that the printer is having difficulties with accurately printing each point of the design in the correct area. Therefore, it can be concluded that 400 μm would be the smallest size of needle that could be printed with a definitive shape at a resolution of 0.025 mm using this printer. However, insertion capabilities would need to be evaluated to ensure that the needles would be able to insert into the skin, as there is a visible reduction in the tip sharpness of the needles in the images shown. This test does provide insight into the size of bores and other shapes that can be printed with this printer, for which sharpness is not a major factor.

### 3.3. Parafilm Insertion Tests

**Figure 5:**
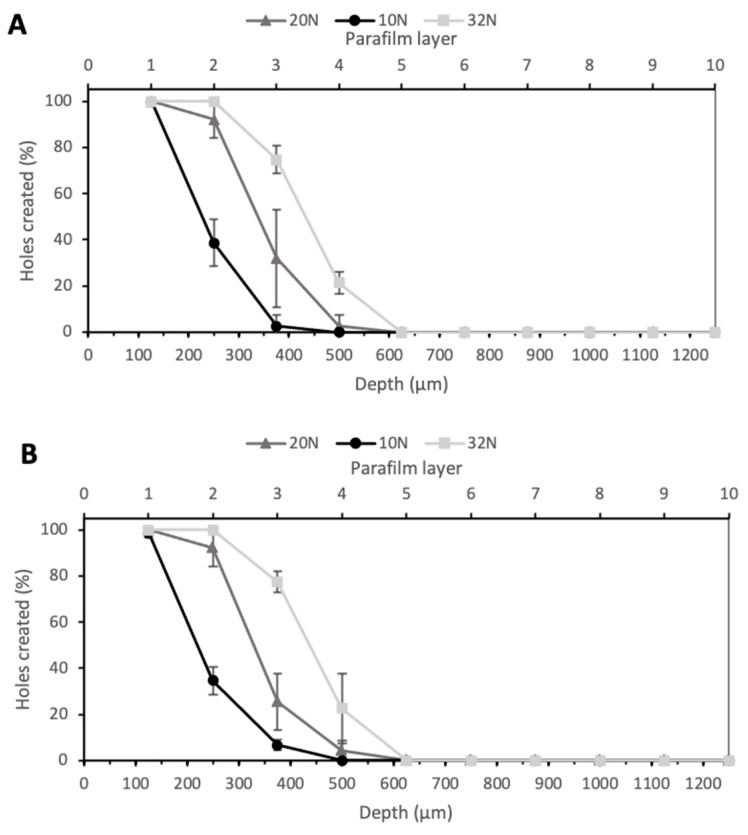
Percentage of holes created in Parafilm layers at 10, 20, and 30 N for PyMN (**A**) and CoMN (**B**).

Larrañeta et al. proposed Parafilm^®^ M as an alternative to biological tissue to perform microneedle insertion studies [22]. MNs insertion ability was investigated at three different forces—10 N, 20 N, and 32 N—as shown in Figure 5. The value 10 N was chosen as the minimum force of insertion tested, as a previous study proved this to be the minimum force at which significant differences in insertion depth could be observed between membranes, while 32 N was used as the higher value as this was the average force of insertion by a group of volunteers in this study; therefore, if MNs could penetrate the Parafilm^®^ M at lower forces, they should be able to bypass the SC layer upon insertion into skin [22]. As expected, an increase in the force led to an increase in the insertion depth. In particular, the arrays with PyMN were able to pierce two layers when an insertion force of 10 N was applied, three layers with a force of 20 N and four layers with 32 N. CoMN, at a force of 10 N, reached the second Parafilm layer but also created a few holes in the third layer (Figure 5B). An increase in the force applied up to 20 N enabled the needles to reach the third layer, leaving a few holes in the fourth; when a force of 32 N was applied, four Parafilm layers were pierced. At 32 N, 100% of needles penetrated the second layer of Parafilm in both PyMN and CoMN; 75% and 77% of needles penetrated the third layer in PyMN and CoMN, respectively. Using the 32 N average force of MN insertion described by Larraneta et al., these MN arrays would be able to insert to a depth of 400 µm in skin [22]. As the MNs are able to insert to an approximate depth of 400 µm, which is half the height of the needles, it is important to position the bore above 50% height of the needles to ensure their minimal leakage occurring during insertion and delivery of a substance. The insertion at 10 N was significantly lower, with around 40% of needles inserted in layer 2 of both PyMN and CoMN. However, 100% of the needles were able to create holes in the first layer of Parafilm, which would be enough insertion depth to bypass the SC.

**Figure 6:**
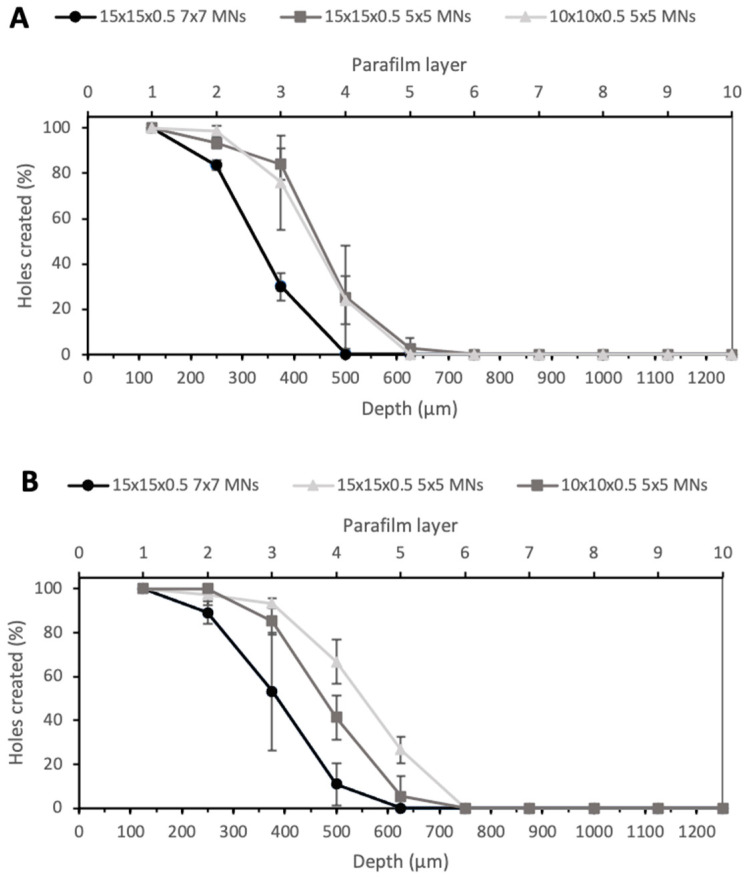
Percentage of holes created in each Parafilm layer by different geometries of PyMN (**A**) and CoMN (**B**) using a force of 32 N.

Another noticeable aspect was that the insertion depth was also dependent on the number of MNs in the array or, more importantly, the spacing between needles on the array. Figure 6 shows the insertion depth obtained for 7 × 7 arrays with PyMN (A) and CoMN (B) at a force of 32 N. The 15 × 15 × 0.5 7 × 7 PyMNs were able to pierce one Parafilm layer less than the 5 × 5 devices with the same MN geometry and showed a significant difference between the numbers of holes created (*p* < 0.05). On the contrary, for the CoMN, the difference in the insertion depth between the 5 × 5 and 7 × 7 arrays was not very significant (*p* > 0.05). When looking at the 5 × 5 needle arrangement on a smaller base plate size of 10 × 10 × 0.5, in PyMN, a similar insertion depth to the 5 × 5 arrangement on a 15 × 15 × 0.5 mm base plate was seen. For CoMN arrays, the smaller base plate size resulted in a slightly lower number of holes created in the third layer in comparison with the 15 × 15 × 0.5 mm base plate. This shows that the when the needles had greater spacing between them, such as in the 5 × 5 arrangement, the MN arrays were able to insert to a higher insertion depth than needles that were spaced more closely together. Therefore, to ensure the optimal insertion capabilities of the MN arrays, a 15 × 15 × 0.5 mm base plate with 5 × 5 needles was chosen for further studies.

### 3.4. Print Angle Optimisation

**Figure 7:**
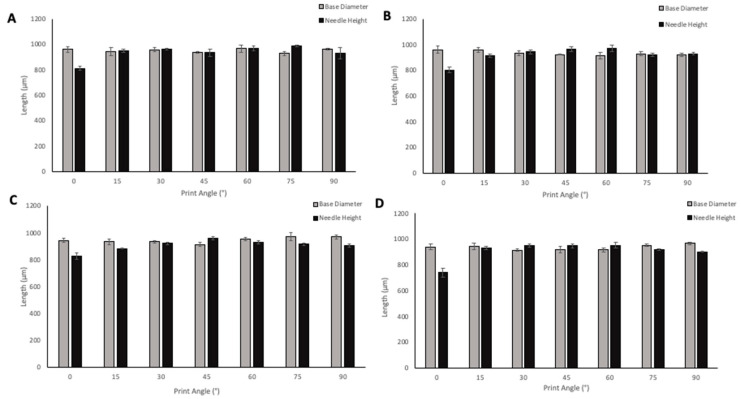
The effect of angle of print on needle height and base diameter of Solid PyMN (**A**), Solid CoMN (**B**), Hollow PyMN (**C**), and Hollow CoMN (**D**).

MNs were oriented at angles ranging from 0–90° to the build plate in order to evaluate the effect of print angle on needle geometries. The size of supporting structures required for printing increased from 0–45° angle prints, which also resulted in an increased print time. A 0° angle of print required 38 min to print the MN array with the possibility to print three replicates in one print cycle; 45° angle required 2 h 17 min to print three replicates of the MN arrays; 60°, 75°, and 90° angled prints required fewer supports than the lower print angles, however, print time still increased due to more layers being required to print the arrays at the higher angles, with 90°-angled arrays requiring 3 h to print. Although increasing numbers of supporting structures were required for some angles of prints, the removal of the supporting structures remained relatively simple. When adding supports, the diameter of the touchpoint at which the supports meet the print could be defined. For all of the prints, the touchpoint size was small; therefore, supports could be easily removed without damaging the needles on the array. Removal of supporting structures from the printed MN is an additional step that adds on some time, as precision is needed to ensure the needles are not damaged; the same risk is present in the demoulding process of MN arrays from the micromoulding method of fabrication. The effect of print angle on needle height and base diameter is shown in Figure 7. When looking at the solid PyMN and CoMN, the print angle that produced needles closest to the design geometry of 1000 µm for PyMN was 75° and for CoMN 60°. When looking at base diameters, 60° in the PyMN and 15° in the CoMN solid produced prints closest to the design geometry. For hollow MNs, needle heights with the closest geometry to 1000 µm were the 45° print in PyMN and 60° in the CoMN. For base diameter, the 90° angle prints produced needles with geometries closest to the design in both PyMN and CoMN designs.

**Figure 8:**
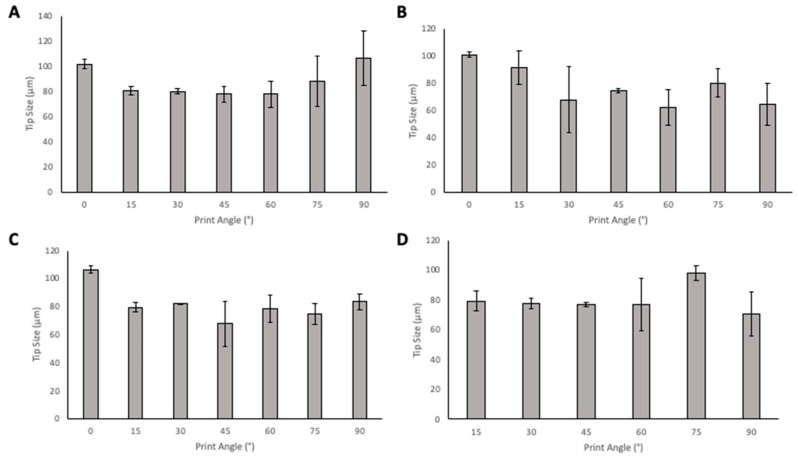
The effect of angle of print on tip size of Solid PyMN (**A**), Solid CoMN (**B**), Hollow PyMN (**C**), and Hollow CoMN (**D**).

When considering tip size (Figure 8), in the solid designs, the 45° angle has the smallest tip size in PyMN with 60° having a similar tip size. In CoMN, a 60° print angle produced needles with the lowest tip size. In the hollow MN designs, for PyMN, 45° print angle produced the smallest tip size and in CoMN design, the 60° print angle produced the lowest tip size with 45° angle having a very close average value to the 60°.

Tip size is a critical parameter to consider when evaluating the printed MNs, as it is essential for determining the sharpness of the needles and a sharper needle leads to better penetration and less pain in patients. When looking at tip size alone, 45° and 60° print angles produced needles with the smallest tip size and, therefore, the sharpest tip needles. Then, considering the needle heights at these angles, 60° print angle produced needles with print heights closer to 1000 µm in PyMN and CoMN solids as well as hollow CoMN. The 45° print angle produced more optimal needle heights in PyMN. Visually, when observing the needles, the 45° angle looked sharper; these needles were also less prone to tip breakages and errors during printing. This may be due to the larger number of supports that are required to print at a 45° angle in comparison with the 60° angle. As supporting structures are printed first, the larger number of supports present protects the print from the detachment process from the tray between each layer of printing. At 60° angle, due to the fewer number of supports, the stress of detaching from the tray is spread out over a smaller number of supporting structures, resulting in a greater chance for fractures and damage during the printing process to the array. The 45° print angle was therefore chosen for future prints to ensure optimal print quality. This is supported by various other articles in which 45° is the angle used for printing with resin-based printers [20,24,25,26]. Although printing at 45° angle presented a high print time of 2 h 17 min in comparison with prints at 0°, when considering the post-printing processes and curing time, this process is still faster than moulding methods used currently, which can take over 24 h for the fabrication of MN arrays.

### 3.5. Mechanical Testing

**Figure 9:**
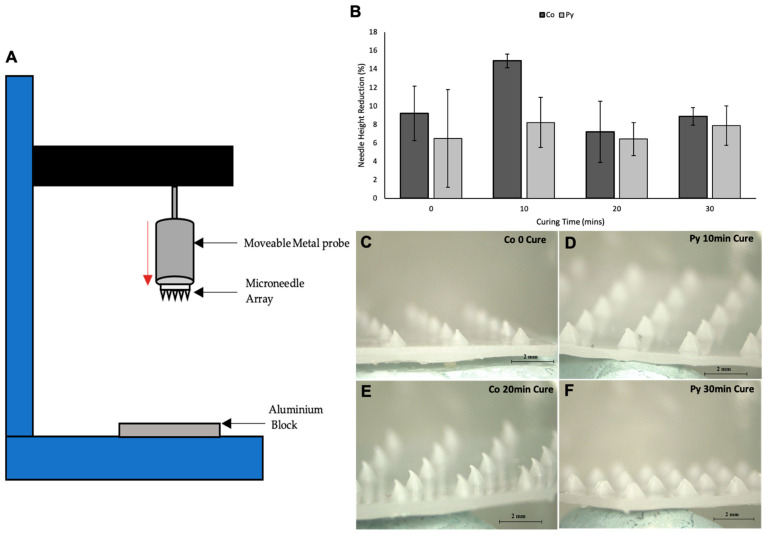
Set up of texture analyser for mechanical testing of MN arrays (**A**). Graph showing the percentage reduction in needle height after mechanical testing of CoMN- and PyMN-shaped arrays (**B**). Light microscope images after mechanical testing of CoMN 0 min cure (**C**), PyMN 10 min cure (**D**), CoMN 20 min cure, and (**E**) PyMN 30 min cure (**F**) MN arrays.

Skin is an elastic part of the body, which possesses significant tensile strength. Therefore, the MNs should be capable of penetrating the skin and SC layer without fracture or breakage of needles occurring [27]. MN arrays were exposed to forces of up to 300 N to determine the fracture force of the MN arrays using the texture analyser set up shown in Figure 9A. Mechanical strength of the MNs are an important factor to consider as it will determine if the MN arrays can withstand the force of insertion. All MN arrays failed in a bend shape rather than fracture, as shown in Figure 9C–F. This shows that MNs did not fracture at forces of up to 300 N. This could mean that the needles are softer rather than fragile as they did not fracture but instead were bent. As no dip in the force displacement curve of the mechanical testing was present, this shows that neither the needles nor base plate fracture under such high forces. As MNs were able to withstand forces of up to 300 N, it can be concluded that MNs should be able to withstand the insertion force into skin without fracture, as average insertion force has been shown to be 32 N in a previous study [22]. Additional Imaging of MN arrays before and after mechanical testing are presented in Supplementary Information for PyMN (S2) and CoMN (S3). Pressing of the MN array was against an aluminium block, which does not directly replicate the insertion of MNs into the skin surface; therefore, this test is only an estimation of the strength of the MN arrays. Overall, the MN arrays displayed good mechanical strength, being able to withstand up to 300 N without fracture and approximately < 15% height reduction in most needles. When comparing the reduction in needle heights between different curing times of the prints, it can be seen that 20 min cure time had the smallest height reduction in both CoMN and PyMN before and after mechanical testing. No curing in the MN arrays produced needles with a large height reduction after mechanical testing in CoMN needles. This could be due to the needles being softer from a lack of curing, leading to the needle tips being crushed under the exerted force. With PyMN with no curing, a lower height reduction of 6.5% was observed; however, there was a large variation in height reductions between PyMNs, meaning the mechanical strength of these needles varied (Figure 9B). A cure time of 10 min produced MN arrays with the greatest height reduction after mechanical testing, with a greater height reduction present in PyMN of 14.9% in comparison with 8.2% in CoMN. Curing of MNs for 30 min produced needles with good mechanical strength, with both CoMN and PyMN having height reductions < 10%; however, the height reduction was lower in 20 min-cured needles. Although the majority of needles bent and did not result in fracture for the 30 min-cured needles, and as can be seen in Figure 9F, some tips were damaged on PyMN resulting in the higher height reduction; as fracture was not present during the exertion of force onto the MN arrays, bent tips could have fallen off during the detachment process from the aluminium block when the probe was being raised. It can be concluded that 20 min would be the optimal cure time for the MN arrays to have optimal mechanical strength without the breakage of brittle needles from overcuring.

## 4. Conclusions

This study has presented a novel approach to the DLP printing of hollow MN arrays. Arrays with sharp MNs that could penetrate Parafilm M layers were produced. MNs presented good mechanical strength and resistance to compression as no fracture was present during mechanical testing. This verifies the appropriate geometries for creating MN arrays with good mechanical strength. This shows the potential for hollow MNs to be created by DLP technique. DLP allows for personalized delivery by altering designs easily using online software; the same design software can be used for all forms of 3DP, allowing for easy transfer of designs. This paper explored some of the critical parameters for optimal print quality. It was found that an angled print produced needles with more optimal geometries than a flat print at 0° to the base plate, with 45° producing the best needles. Prints cured for 20 min also performed the best during mechanical testing with the smallest reduction in height after testing. However, as this is a novel approach, there are areas that need further investigation before it can be brought to market. Biocompatibility testing of the materials used is necessary to ensure that commercial material is suitable for application to the skin. Use of novel biocompatible resins, created in the lab using a combination of polymers and photoinitiators, would increase the range of applications of this technique. However, on the basis of proof of concept for printing with the MN system, this study proves great potential for future applications of Hollow MN arrays using 3D printing with a faster approach to fabrication than current methods.

## Figures and Tables

**Table 1:** Summary of three resin-based printers and their advantages and disadvantages.

	Features	Advantages	Disadvantages
DLP	Digital projector flashes single image of each layer over build platform.	Faster than SLA as full layer is projected onto tray each time. Less material waste for small prints.	Smaller resin tank so not suitable for large prints. Vertical voxel lines created.
SLA	UV laser combined with galvanometers to direct laser beam across print area.	Suitable for large prints due to presence of larger resin tanks. Wide range of compatible resins available. Smooth print finish.	Slower than DLP and LCD as laser needs to scan through each layer.
LCD.	LED array Light source in combination with an LCD photomask.	Fast printing. Cheap.	Lower print quality than SLA and DLP. Vertical voxel lines visible.

## Data Availability

Data available on request due to restrictions.

## Supplementary Materials

The following are available online at https://www.mdpi.com/article/10.3390/pharmaceutics13111837/s1, Figure S1: Showing Solid MN prints of 800 µm CoMN (A), PyMN (B), 600 µm CoMN (C), PyMN (D), 400 µm CoMN (E), PyMN (F), and 200 µm CoMN (G) and PyMN (H), Figure S2: Optical Microscopy Images of PyMN arrays before and after mechanical testing, Figure S3: Optical Microscopy Images of CoMN arrays before and after mechanical testing.
