# Supplementary material for: Optimization of Printing Parameters for Digital Light Processing 3D Printing of Hollow Microneedle Arrays
Source: Pharmaceutics. 2021 Nov 2;13(11):1837. doi: 10.3390/pharmaceutics13111837 (PMC8622592; doi:10.3390/pharmaceutics13111837)
Supplement: Supplementary file 1 [file pharmaceutics-13-01837-s001.zip › pharmaceutics-1386531-supplementary.pdf]

pharmaceutics

MDPI

Supplementary Materials: Optimization of Printing Parameters for Digital Light Processing 3D Printing of Hollow Microneedle Arrays

Essyrose Mathew, Giulia Pitzanti, Ana L. Gomes dos Santos and Dimitrios A. Lamprou

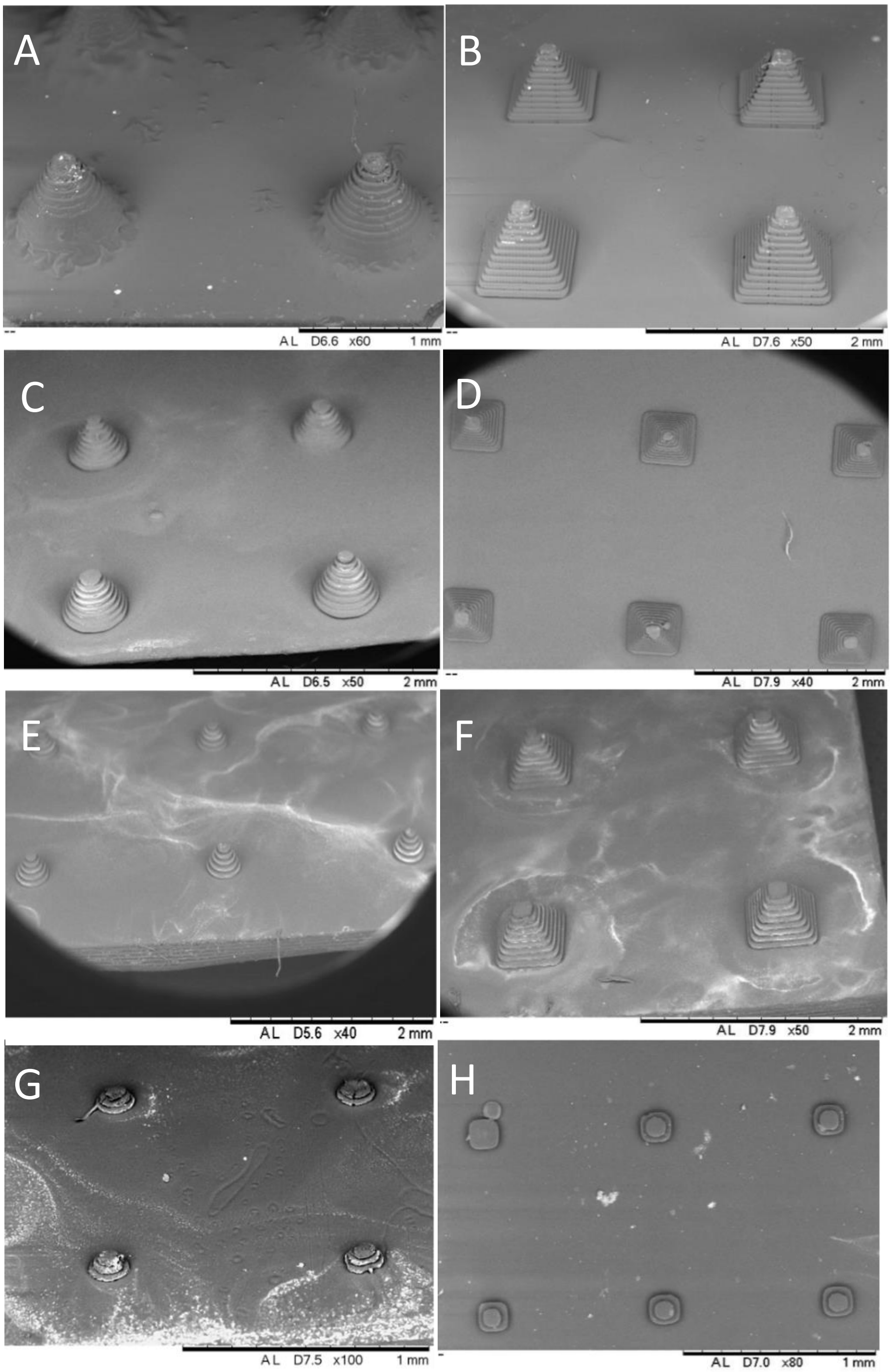

Figure S1. Showing Solid MN prints of 800 µm Co (**A**), Py (**B**), 600 µm Co (**C**), Py (**D**), 400 µm Co (**E**), Py (**F**) and 200 µm Co (**G**), Py (**H**).

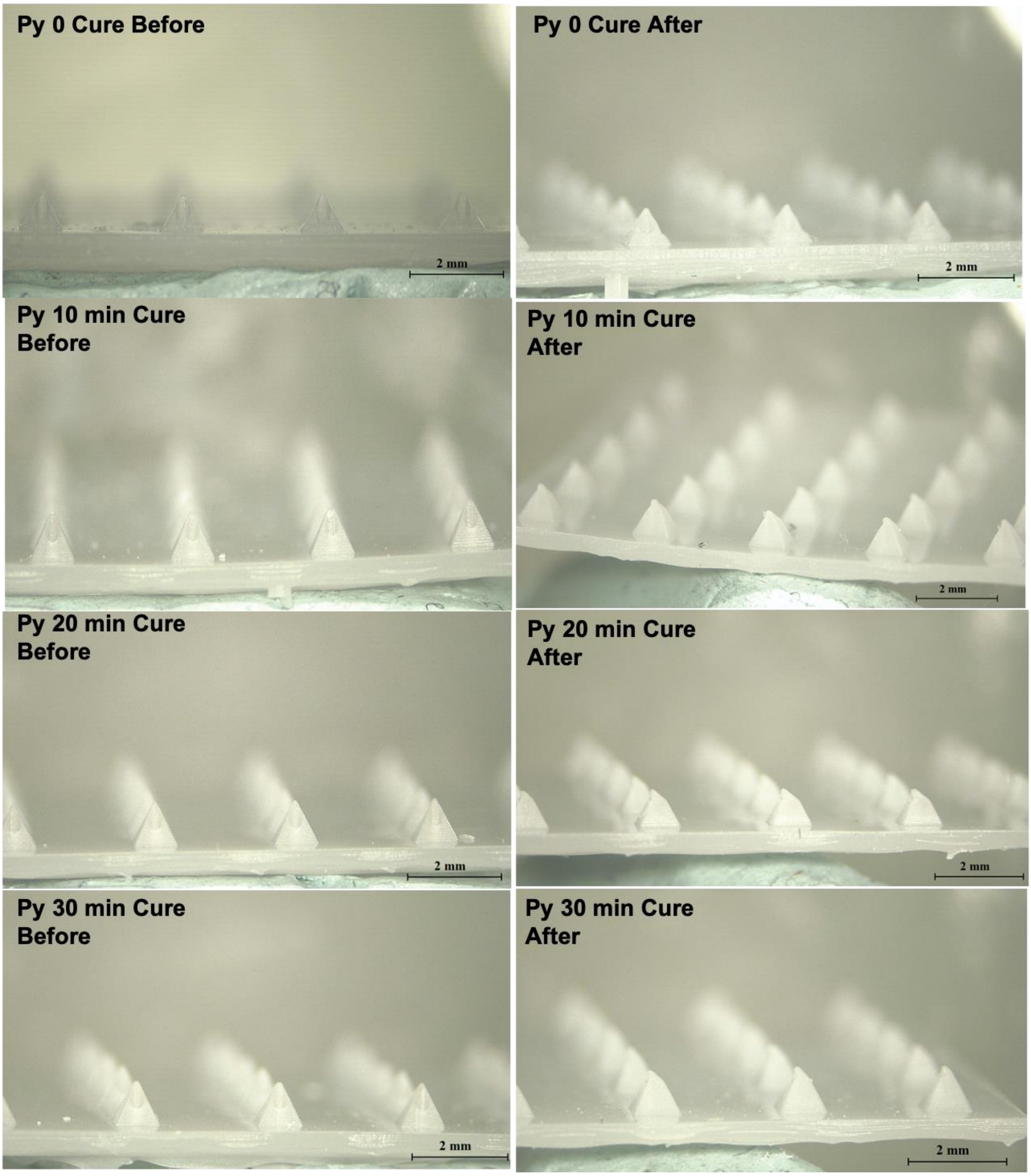

Figure S2. Optical Microscopy Images of Pyramidal MN arrays, before and after mechanical testing.

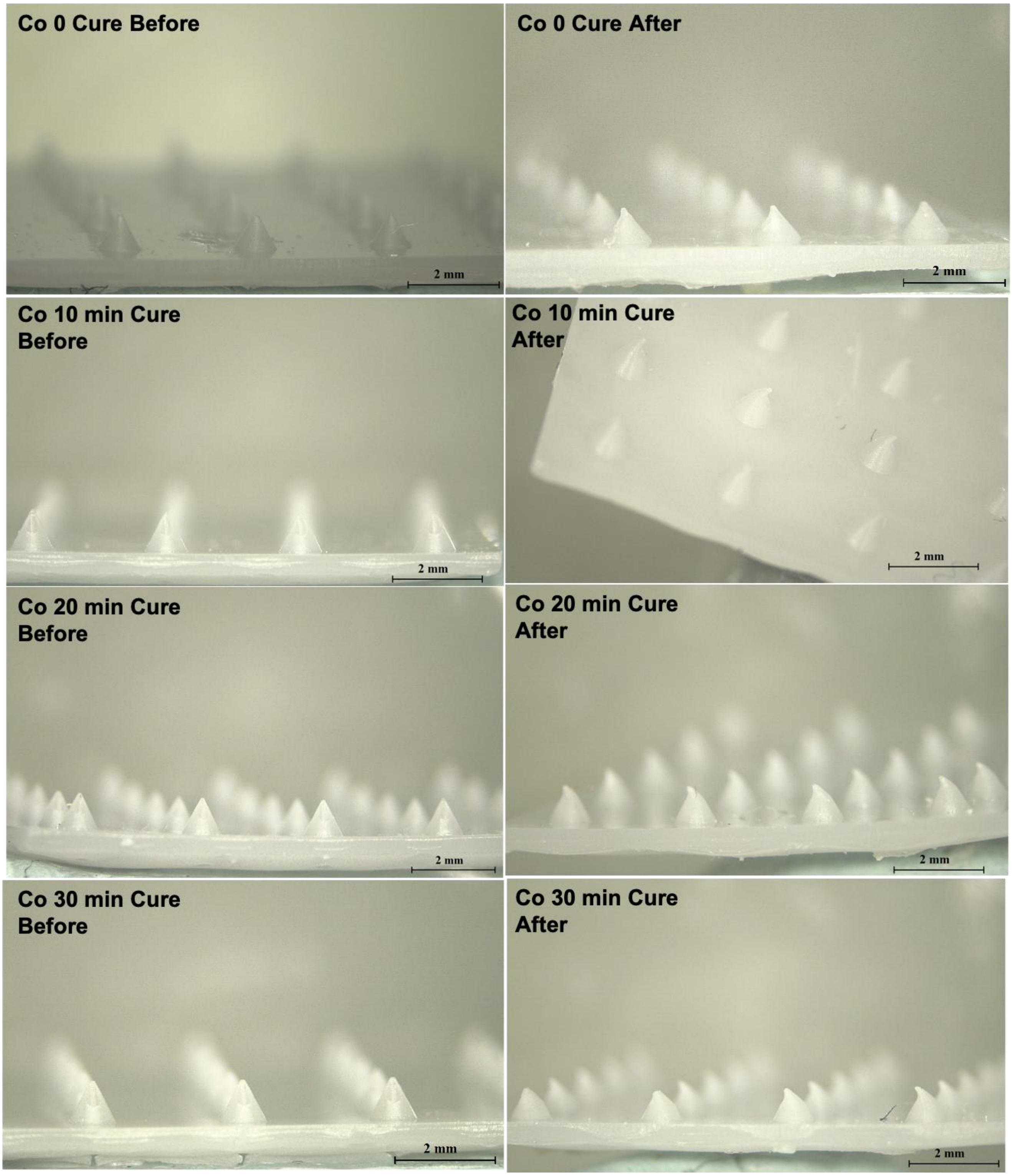

Figure S3. Optical Microscopy Images of Conical MN arrays, before and after mechanical testing.
